# Conservative Mechanisms of Extracellular Trap Formation by Annelida *Eisenia andrei*: Serine Protease Activity Requirement

**DOI:** 10.1371/journal.pone.0159031

**Published:** 2016-07-14

**Authors:** Joanna Homa, Weronika Ortmann, Elzbieta Kolaczkowska

**Affiliations:** Department of Evolutionary Immunology, Institute of Zoology, Jagiellonian University, Gronostajowa 9, 30-387, Krakow, Poland; The Hospital for Sick Children and The University of Toronto, CANADA

## Abstract

Formation of extracellular traps (ETs) capturing and immobilizing pathogens is now a well-established defense mechanism added to the repertoire of vertebrate phagocytes. These ETs are composed of extracellular DNA (extDNA), histones and antimicrobial proteins. Formation of mouse and human ETs depends on enzymes (i) facilitating decondensation of chromatin by citrullination of histones, and (ii) serine proteases degrading histones. In invertebrates, initial reports revealed existence of ETs composed of extDNA and histones, and here we document for the first time that also coelomocytes, immunocompetent cells of an earthworm *Eisenia andrei*, cast ETs which successfully trap bacteria in a reactive oxygen species (ROS)-dependent and -independent manner. Importantly, the formation of ETs was observed not only when coelomocytes were studied *ex vivo*, but also *in vivo*, directly in the earthworm coelom. These ETs were composed of extDNA, heat shock proteins (HSP27) and H3 histones. Furthermore, the formation of *E*. *andrei* ETs depended on activity of serine proteases, including elastase-like activity. Moreover, ETs interconnected and hold together aggregating coelomocytes, a processes proceeding encapsulation. In conclusion, the study confirms ET formation by earthworms, and unravels mechanisms leading to ET formation and encapsulation in invertebrates.

## Introduction

Over 95% of animal species are invertebrates and all of them utilize only one arm of immunity, the innate response [1,2]. This fact itself affirms importance of innate immunity but paradoxically, we know much more about vertebrate mechanisms (although often homologous to invertebrates) than those operating in lower taxa. In line with a new model of Sequential Immune Responses (SIR) [3] earthworms represent invertebrate animals with SIR1 and SIR2 i.e. for defense they use rapidly activated enzymes such as NADPH oxidase generating reactive oxygen species (ROS) [4] and have macrophage-like immunocompetent coelomocytes [5], respectively. Immunocompetent cells of earthworms are called coelomocytes and can be divided into amoebocytes and eleocytes [6–8]. Both types of coelomocytes can recognize foreign materials (e.g. pathogens) and perform phagocytosis and encapsulation [9–11]. Coelomocytes function in the coelom where immune responses take place. In fact earthworms also possess characteristics of SIR3 as recently expression of bacteria-sensitive TLRs was confirmed on coelomocytes [12,13], and the cells also release diffusible nitric oxide (NO) [14,15].

One of the highlights of SIR2 is evolvement of neutrophils and neutrophil-like cells with even more profound ROS-dependent killing and formation of extracellular traps (ETs) [3]. Originally formation of such ETs was reported in mammals and was attributed particularly to neutrophils, hence named *neutrophil extracellular traps* i.e. NETs [16]. NETs belong to ETs (being neutrophil ETs) and are released by highly activated neutrophils, when phagocytosis and/or release of antimicrobials by degranulation are not sufficient any more to contain infection or the pathogen is too big. In such circumstances neutrophils release structures which backbone is made up by DNA (of nuclear or, rarely, mitochondrial origin) to which granular, cytosolic and nuclear proteins are attached [16,17]. NETs are aimed to capture, immobilize and frequently kill pathogens [18]. The proteins attached to NETs include histones, proteases (e.g. neutrophil elastase, cathepsin G), oxidative enzymes (e.g. myeloperoxidase, MPO) and antimicrobial proteins such as lactoferrin [19,20]. It should be underlined that histones are the main protein components of chromatin that compact and help condensate DNA and also possess antimicrobial properties [16].

What makes NETs/ETs really unique is a mechanism of their formation. In the mammalian system, two enzymes are critical for NET formation, serine protease neutrophil elastase (NE) and protein arginine deiminase/peptidyl arginine deiminase type IV(PAD4).While PAD4 citrullinates histones [21], NE is believed to degrade them [22]. It has been proposed that histone citrullination promotes a relaxing of the chromatin structure, allowing NE to gain access to histones resulting in promotion of nuclear decondensation [23]. Interestingly, also other serine proteases (including cathepsin) were shown to act in a similar manner to NE i.e. their binding to DNA/RNA promotes nuclear localization and cleavage of nucleic acid binding proteins, including histones [24]. On the other hand, PAD4 activation in neutrophils seems to require cytoskeletal activity as it can be suppressed, along with NET formation, by an inhibitor of actin polymerization, cytochalasin D [25].

Subsequently, also other mammalian leukocytes were shown to form ETs, namely macrophages [26] and eosinophils [27], and then non-mammalian vertebrate species were reported to release ETs, e.g. [28]. Furthermore, three groups reported of ETs being released by seawater invertebrates: shrimp *Litopenaeus vannamei* hemocytes [29,30] and shrimp *Marsupenaeus japonicus* [31], oyster *Crassostrea gigas* [32], shore crab *Carcinus maenas*, blue mussel *Mytilus edulis* but also by sea anemone *Actinia equine* [33]. Especially the data on *A*. *equine* is appealing as it indicates that release of ETs is primordial and predates the evolution of the coelom and thus could be considered as an additional SIR1 mechanism [33]. Just recently, the evolutionary conservatism of casting ETs was further confirmed in the social amoeba *Dictyostelium discoideum*. In this species, sentinel cells of the multicellular slug stage of the amoeba successfully form ETs in response to bacterial components [34].

The release of extracellular DNA (extDNA) does not have to be indicative of NETs/ETs, and in fact reports on free circulating DNA in the circulatory system of man, now largely attributed to disintegrating NETs, were neglected for decades only because they were attributed to necrotic cells [35]. Thus NETs/ETs are defined only when extDNA is decorated with histones and cytoplasmic/granular proteins. The first reports on ETs in invertebrates focused on presence and anti-microbial functions of extracellular DNA (extDNA) itself [18], and the two following papers incorporated also data on presence of histones attached to extDNA [18,21] but no other, non-enzymatic protein components of invertebrate ETs were reported so far. Moreover, mechanisms of invertebrate ET formation are not know. In particular, involvement of proteases has not been studies so far. However, proteases do exist in earthworms and in fact serine proteases, including elastase-like proteases were also described in Annelida [36].

The process of citrullination has not been studied in invertebrates, and PADs were not reported to be present in other animals than vertebrates, except of bacteria *Porphyromonas gingivalis* [37]. However, cytochalasin D was shown to inhibit ET release in the shore crab *C*. *maenas* [33].

One of the killing mechanism common to all animals (SIR1) is generation of ROS which are highly cytotoxic and thus antimicrobial agents [3,4]. Apart of this function, however, they can also act as reversible signal transduction mediators to regulate redox-sensitive target proteins [25]. Dependence of NET formation on generation of ROS was repeatedly reported, e.g. [38,39]. However, it should be mentioned that NET formation is not always ROS-dependent and in fact it might depend on the timing or stimulus, with the bacterial components acting commonly independently of ROS, e.g. [17,39,40].

The aim of the studies presented here was to verify ET formation by a representative of Annelida (*Eisenia andrei*), in which ET release was not yet studied, as well as to elucidate mechanisms of ET formation in non-vertebrate species. In particular, to verify if earthworms release ETs when fighting infection in their coelom, we treated the animals with immunostimulants and imaged microscopically ET formation inside of the coelom (the *in vivo* setting). To quantify release of ETs, we isolated coelomocytes from immunostimulated individuals, and after culturing evaluated ET release *ex vivo* (the *ex vivo* setting). Finally, in order to study mechanisms of ET formation, we isolated coelomocytes from untreated animals, immunostimulated them *in vitro* simultaneously modulating the ET release by different inhibitors and active compounds (the *in vivo* setting).

Here we report that similarly to vertebrate NETs, earthworm ET formation also depends on serine protease-activity and that histones H3 are attached to ETs. We also identified some of the components of earthworm ETs (other than extDNA) and showed capacity of ETs to capture bacteria. We also demonstrated connection between ET formation and the process of encapsulation, and document ET formation *in vivo* by imaging the traps inside of *E*. *andrei* coeloma. Therefore, the current study adds Annelida and its exemplary terrestrial species to the list of invertebrates capable of ET release but most importantly provides insides into innate mechanisms of ET formation in lower animal taxa.

## Materials and Methods

### Experimental animals

Adult (clitellate) earthworms (0.45–0.75 g body weight) of *Eisenia andrei* (Sav.) were collected from the stockbreeding maintained in the Institute of Zoology of the Jagiellonian University, kept in controlled laboratory conditions (20±1°C; 12:12 LD) in commercial metal-free soil (PPUH Biovita, Poland).

### Experimental setting

Experiments were performed in 3 settings: *in vivo*, *ex vivo* and *in vitro*. In (i) the *in vivo* setting, immunostimulated animals (details are described below; some individuals were pre-treated with diverse inhibitors prior to immunostimulation) were used for microscopic observations of their coelom. In (ii) the *ex vivo* setting, coelomocytes were isolated from some of the immunostimulated animals, and used to evaluate ET release outside of the body, both quantitatively (as described in *Quantification of extracellular DNA released by stimulated coelomocytes*) and by immunostaining. In (iii) the *in vitro* setting, coelomocytes were isolated from some unstimulated earthworms and immunostimulated *in vitro* to study mechanisms of ET release. Because of this, coelomocytes were treated with differential inhibitors and/or active compounds prior to immunostimulation. Subsequently, respiratory burst of the cells was measured as well as release of extDNA (*Quantification of extracellular DNA released by stimulated coelomocytes*).

In detail, in (i) the *in vivo* studies animals were injected with 20 μl of immunostimulants into the coelomic cavity (1 cm behind the clitellum): phorbol 12-myristate 13-acetate (PMA, Sigma-Aldrich, 0.16 nMol i.e. 0.1 μg/ml) and *Xenorhabdus bovienii* (BACT, 5x10^7^ CFU/ml). Control (CTR) animals were injected with saline solution (0.9% NaCl). Twenty four hours after injections, the earthworms were used for microscopic imaging. In (ii) the *ex vivo* studies, earthworms were firstly injected with immunostimulants: PMA (0.16 nMol i.e. 0.1 μg/ml); *X*. *bovienii* (BACT, 5x10^7^ CFU/ml); lipopolisaccharide (LPS lyophilized cells, resuspended in saline, Sigma-Aldrich, 1 mg/ml); *Micrococus luteus* (M.l, Sigma-Aldrich, 1 mg/ml); zymosan A (Z, from *Saccharomyces cerevisae*; Sigma-Aldrich, 1 mg/ml); hydrogen peroxide (H_2_O_2_, Sigma-Aldrich, 100 mM); control (CTR) animals were injected with saline solution (0.9% NaCl, Baxter Terpol, Poland). After injections, the animals were placed individually in 15 ml vials filled with filter paper soaked with water, modified [15]. Coelomocyte isolation took place after 24 hours since the stimulation, and ET formation was observed in culture conditions, in slide chambers after following 24 hrs. In (ii) the *in vitro* studies, coelomocytes were isolated from untreated animals and stimulated *in vitro* with 10 μl of PMA (0.16 nMol i.e. 0.1 μg/ml) and BACT (5x10^7^ CFU/ml), LPS (10 μg/ml), M.l (100 μg/ml), Z (100 μg/ml) or H_2_O_2_ (100 mM). Coelomocyte monolayers (1x10^6^ cells/ml; 10^5^/well) were stimulated with the above compounds for 1–24 hours i.e. formation of ETs was monitored after 1, 2, 4, 6 and 24 hours.

### Harvesting of coelomocytes

In order to isolate coelomocytes, the earthworms were stimulated for 1 min with a 4.5 V electric current to expel coelomic fluid with coelomocytes through the dorsal pores according to the procedure described previously [41]. Coelomocytes were collected in Sörensen solution (0.05 M; Na_2_HPO_4_/KH_2_PO_4_, pH 7.4) and seated at 1x10^6^/ml (10^5^/well) in slide chambers (Haimen Changlong Instrument Co., China) (*in vitro* and *ex vivo* settings as described in section *Experimental setting*). In all experiments, coelomocytes were collected and analysed individually from each earthworm without cell pooling.

### Bacteria culture and staining

*X*. *bovienii* were isolated from nematode *Steinernema feltiae*. The bacterial stockbreeding culture was a gift of Dr. Paulina Kramarz from Institute of Environmental Sciences (Jagiellonian University). Bacteria were propagated in YS broth in 100-ml Erlenmeyer flasks filled with 30 ml medium containing (in g/l^−1^) 5 NaCl, 5 yeast extract, 0.5 NH_4_PO_4_, 0.5 K_2_PO_4_, 0.2 MgSO_4_×7 H_2_O (Merck) at 180 rpm and 25°C. Subsequently, bacteria were plated onto agar plates for 48 h in 25°C. Then CFUs were counted and collected, and their aliquots were prepared and frozen. Before addition of *X*. *bovienii* to coelomocytes, bacterial cultures were transferred into Eppendorf tubes, washed and resuspended in saline [42].

Capacity to capture bacteria by ETs was assessed by fluorescent microscopy (some *ex vivo* and *in vivo* experiments). For this, *X*. *bovienii* were stained with 10 μM Sytox green (volume proportion 1:1, Molecular probes, Eugene, OR) for 30 min at room temperature. The bacterial suspension was then pelleted by centrifugation (3000 rpm, 5 min, RT) and washed and resuspended in saline. Bacteria were either injected into *E*. *andrei* or added to the coelomocyte cultures as described in section *Experimental setting*. Sytox dyes were showed to stain both live and dead bacteria and do not affect bacterial growth [43].

### Quantification of extracellular DNA released by stimulated coelomocytes

The release of DNA from coelomocytes in response to different stimuli was determined according to [28] in the *in vitro* setting (as in section *Experimental setting*). The amount of extDNA was assessed by Sytox dyes which selectively bind DNA in a process that involves electrostatic interactions, by intercalating (inserting molecules between the planar bases of DNA) cooperatively with a 3.5-bp binding site size [44]. DNA release was measured in coelomocytes after 24 hour cell incubation with stimulants (PMA, BACT, LPS, M.l, Z). The 10 μl of Sytox orange (5 μM, Molecular Probes, Eugene, OR) was added to the cell cultures (1x10^6^ cells/ml; 10^5^/well) in a 96-well plates, and after 5 min the fluorescence was determined as arbitrary fluorescence units (AFU, excitation 547 nm, emission 570 nm) using a microtiter plate spectrofluorometer (SpectraMax M5, Molecular Devices, USA) with SoftMax Pro Software (v 5.4.4.007).

### Detection of coelomocyte extracellular traps (ETs)

Fluorescence microscopic imaging of ETs was performed with Sytox orange (10 μl of 5 μM). Immunostimulated earthworms (as in *Experimental setting*) were firstly placed on ice for 10–15 min to allow easy dissection because of muscle relaxing (the *in vivo* setting). Subsequently, an incision was performed on the dorsal side of the earthworm behind clitellum to open the body cavity, which allowed for microscopic imaging of the coelom. Sytox orange was directly added to the coelomic fluid. In the *ex vivo* and *in vitro* settings, Sytox orange was added to the coelomocyte cultures, and after 5 minutes the extracellular DNA was stained with the dyes. In some experiments acridine orange (10 μM, ThermoFisher Scientific) was used to differentially stain for nucleated cells. Then microscopic observations were performed and images were obtained with a fluorescence microscope Zeiss AxioImager. M2 equipped with AxiocamMRm (monochrome CCD) camera and analyzed using the image processing with the AxioVision Imaging System (Based on Release 4.8). For each treatment representative images were obtained and are presented in this work. None of the solvents used to prepare compounds tested in the study (DMSO, ethanol) did not affect ET formation when applied in the amount corresponding to the one used to dissolve the drugs. The inhibitors themselves did not induce cell toxicity.

### ET treatment with DNase and heparin

To verify specificity of Sytox staining, DNase dissolving DNA which serves as a backbone of ETs was used [16]. Fresh medium containing 10 U DNase I (EURx Molecular Biology Products, Poland) was added to the wells containing coelomocytes (the *ex vivo* or *in vitro* settings). Some ETs were also treated with heparin (100 μM; Sigma-Aldrich) which has high affinity for histones and releases them from chromatin thus disintegrates ETs [45].

### Immunostaining (H3, H3Cit, HSP27)

ETs were identified as extracellular structures that were simultaneously immunoreactive for DNA and histone H3 (H3) or citrullinated histone H3 (H3Cit). Coelomocytes were obtained according to either *ex vivo* or *in vitro* protocols (section *Experimental setting*). The slides were fixed with cold 70% ethanol, air-dried and frozen (-20°C). Just before the immunofluorescence staining, the slides were washed with PBS and then blocked with 3% BSA in PBS for 45 min at room temperature and then incubated overnight at 4°C with either of primary antibodies: Anti-Histone rabbit polyclonal synthetic antibody (corresponding to human histone H3) (1:200, H3, ChIP Grade ab1791, Abcam, Cambridge, UK) or anti-Histone H3 citrulline, rabbit polyclonal synthetic antibody (corresponding to human histone H3 aa 1–100 cytrulline R2+R8+R17) (1:200, H3cit, ChIP Grade ab5103, Abcam, Cambridge, UK). After washing with PBS, the cells were reacted for 1 h at room temperature with Cy^™^3-conjugated goat anti-rabbit IgG (H+L), (1:300, 111-165-144, Jackson ImmunoResearch Laboratories Inc.). Appropriate negative controls were run without the primary antibodies.Then the slides were washed again with PBS and counterstained using Sytox orange (5 μM) or Sytox green (10 μM, Molecular Probes) to visualize extDNA.For immunostaining of heat shock protein 27 (HSP27) associated with extDNA, mouse IgG1 anti-Hsp27 monoclonal (clone G3.1) antibody (1:100, ADI-SPA-800, StressGen, Victoria, BC, Canada) and then anti-mouse IgG (H+L) goat polyclonal FITC conjugated (1:300, A90-138F, BETHYL Laboratories, Inc.) were used, respectively. The counterstaining of extDNA was done with Sytox orange (5 μM).

### Coelomocytes extracellular trap inhibition

ETs formation was challenged by several inhibitors (listed below). In the *in vitro* setting coelomocytes were pre-treated for 1 hour with appropriate inhibitors and then with the immunostimulants (as in *Experimental setting*). In the *in vivo* and *ex vivo* settings, inhibitors were injected 1 hour before immune-stimulation (as in section *Experimental setting*).

### Inhibitor of NADPH oxidase

To determine involvement of ROS in ET production, cells were pre-treated with NADPH oxidase inhibitor, diphenyleneiodonium (DPI, 5 and 50 μM, Calbiochem, San Diego, California) in the *in vitro* conditions [28].

### Inhibitor of Autophagy

To determine whether autophagy is necessary for ET release, a typical autophagy inhibitor wortmannin, was used. Wortmannin is a cell-permeable fungal metabolite that acts as a potent, selective and irreversible inhibitor of phosphatidylinositol 3-kinase (PI3K) which is required for autophagy [46,47]. Wortmannin (Sigma-Aldrich) was used in a concentration of 100 nM in the *in vitro* tests [48,49].

### Inhibitors of Cytoskeleton

Cytochalasins are actin polymerization inhibitors impacting not only cytoskeletal structure and cell morphology but also processes that depend on the cytoskeletal changes. In particular, cytochalasine D (Cyto-D, Sigma-Aldrich) was shown previously to inhibit NET release, [25]. Both cytochalasins can also inhibit phagocytosis [50,51] but Cytochalasin B is a stronger phagocytosis inhibitor in the case of earthworm coelomocytes (unpublished observation). *In vitro* they were used in the following concentrations: Cyto-D (5, 20, 100 μM) or Cyto-B (20 μM). In the *in vivo* tests, Cyto-D was applied in a concentration of 20 μM. The dosage of Cyto-D and Cyto-B was according to [25,52].

### Inhibitors of Apoptosis

To test whether extDNA is released as a result of apoptosis, coelomocytes were incubated with a pan-caspase inhibitor Z-VAD-FMK (100 μM, VAD, carbobenzoxy-valyl-alanyl-aspartyl-[O-methyl]- fluoromethylketone, Promega, Corporation, Madison, WI). VAD is a cell-permeant inhibitor that irreversibly binds to the catalytic site of caspase proteases and inhibits induction of apoptosis [53]. To more specifically address this issue, we also applied an inhibitor of a master caspase—caspase 3 (Cas-3, 250 μM, Calbiochem). All tests were run in the *in vitro* experimental setting.

### Inhibitors of Proteases

To investigate whether proteases are required to form extracellular traps in coelomocytes, protease inhibitor cocktail (cOmplete Protease Inhibitor Tablets, Roche) was used as a broad protease inhibitor (BROAD PROT). It inhibits serine, cysteine, and metalloproteases in bacterial, mammalian, yeast, and plant cell extracts (CO-RO, Roche Mannheim, Germany) [54]. Additionally, we used [phenylmethlysulfonyl] fluoride (PMSF, Roche, Mannheim, Germany) that inhibits mainly serine proteases (1 mM, SERINE PROT) [55]. One milliliter of BROAD PROT contains: 375 μg/ml pancreas extract; pronase, 0.375 μg/ml; thermolysin, 0.2 μg/ml; chymotrypsin, 0.37 μg/ml; trypsin, 0.5 μg/ml; papain, 250 μg/ml (Roche, Mannheim, Germany). 10 μl of inhibitors were added to the cells *in vitro* or earthworms (the *in vivo* setting) were injected with 20 μl.

### Inhibitor of Elastase

The activity of elastase was inhibited by a treatment of coelomocytes with a potent neutrophil elastase inhibitor (NEI) Sivelestat (Sigma-Aldrich) [56,57]. *In vitro* sivelestat was used in a concentration of 100 and 500 μM, and *in vivo* of 500 μM.

### Inhibitor of peptidylarginine deiminase/PAD4

Effects of cell-permeable Cl-amidine (Calbiochem), an inhibitor of PAD4 activity, was tested on ET formation by coelomocytes. *In vitro* Cl-amidine was used in a concentration of 20 and 200 μM and *in vivo* of 200 μM [58].

### Respiratory Burst

The respiratory burst activity of coelomocytes was measured with the nitroblue tetrazolium (NBT) as described previously [15]. Suspension of coelomocytes, 1x10^6^/ml (1x10^5^/well) was incubated with NBT (10 mg/ml, Sigma-Aldrich) for 1 h, then the reaction was stopped with methanol. The plates were air-dried and 120 μl of 2 N potassium hydroxide and 140 μl of dimethyl sulphoxide were added to each well. The optical density (O.D.) was recorded in an ELISA reader (Expert Plus, AsysHitach GmbH, Austria) at 540 nm.

### Protease activity—zymography

For detection of active proteases, gelatin zymography was used. Zymography was performed as described previously with some modifications [59]. The method was used to assess protease activity in samples collected from stimulant-treated earthworms (section *Experimental setting*; the *in vivo* settings). Briefly, samples were normalized for protein concentration and electrophoresed in 10% sodium dodecyl sulphate–polyacrylamide gels, containing 1% porcine gelatin (Sigma-Aldrich), under non-reducing conditions. The gels were washed twice in 2.5% Triton X-100 (15 min each) and developed for 48 hours at 37°C in incubation buffer (50 mM Tris-HCl, pH 8.0, 5 mM CaCl_2_, 0.02% NaN_3_ and 1 μm ZnCl_2_). They were fixed and stained with 0.5% Coomassie brilliant blue (Sigma-Aldrich) in acetic acid/isopropanol/distilled water 1:3:6 and then washed in equilibrating solution with 40% methanol, 10% acetic acid and 3% glycerol (all from Sigma-Aldrich) [59]. Gelatin degradation was visualized under long-wave ultraviolet light. Pre-stained broad-range molecular weight standards (Bio-Rad) were used. Densitometric analysis of protein bands was performed with the uvisoft-uvimap program (UVItec, Ltd, Cambridge, UK).

### Statistic

Results are expressed as means + standard deviation (X+SD). On figures, data are recalculated as percentage of control (100%). The level of significance was established at p<0.05. Statistical comparisons of the control and stimulated groups were performed with one way ANOVA (Tukey as a post-hoc). Each experiment was repeated at least 3 times and in each of them 3 to 4 individuals were used.

## Results

### Earthworm coelomocytes form extracellular traps (ETs)

**Fig 1:**
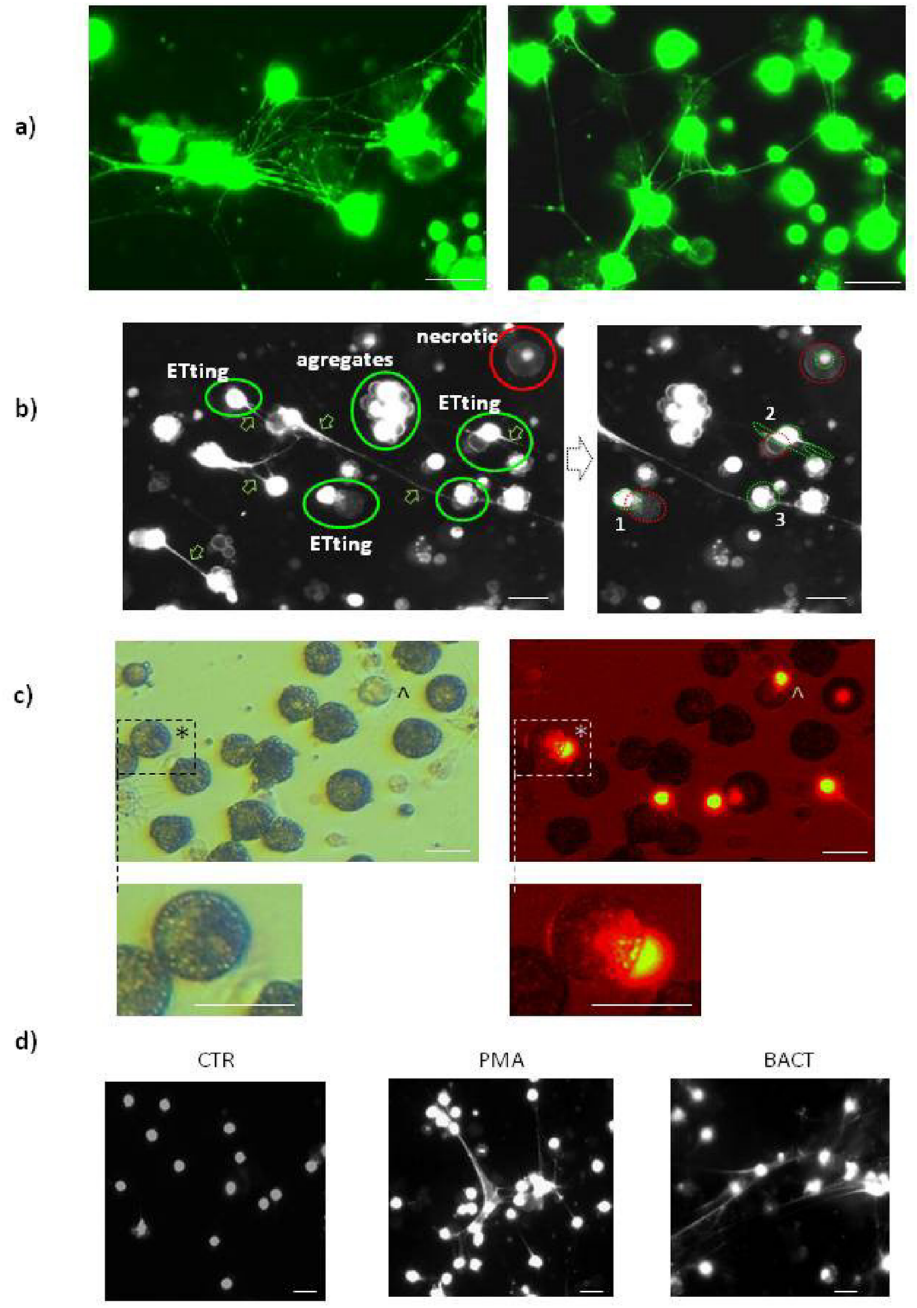
Earthworm *E*. *andrei* coelomocytes form extracellular traps (ETs) composed of extracellular DNA (extDNA). Representative images ofcoelomocytes that released ETs or are in a process of their release (ETting). Coelomocytes retrieved from *E*. *andrei* were seated in slide chambers and stimulated with PMA (unless otherwise stated) and after 24 hours Sytox orange was added to stain extDNA (on some images Sytox orange is shown in false white or green colour for contrast). a) Representative images revealing networks of extDNA (ETs) released by coelomocytes. b) coelomocytes captured at different stages of ET release (exemplary ETs are marked with arrows). As demonstrated on the right hand side image, some coelomocytes are in a process of extruding their DNA (1, 2) while some have already released ETs and are partially covered by extDNA (3). Only few necrotic cells can be seen. Cell bodies are marked with red dotted line while their DNA with green dotted line. c) bright field (left) and fluorescent (right) images of extDNA being released by coelomocytes. Both eleocytes (*) and amoebocytes (^) were captured during this process. Enlarged fragments of images are also presented to show integrity of the exemplary eleocyte releasing ET and the DNA bulb being extruded. d) appearance of extDNA in coelomocyte cultures resulting from preceding stimulation of earthworms with PMA or bacteria *X*. *bovienii* (BACT) *versus* untreated earthworms (CTR).

Coelomocytes isolated from *E*. *andrei* which were stimulated *in vivo* for 24 hours with various stimulants of the immune system, including bacterial (LPS) and fungal (zymosan) components as well as whole live bacteria (Gram^-^*Xenorhabdus bovienii* and Gram^+^*Micrococcus luteus*) released extracellular DNA (extDNA) when kept *ex vivo* (Fig 1 and S1 Fig). In addition, hydrogen peroxide (H_2_O_2_), generated during respiratory burst, also stimulated coelomocytes to form the traps. When quantified (the measurement of extDNA with a fluorescent plate reader), the intensity of extDNA release was comparable to PMA, a well-established ET stimulant, while untreated coelomocytes hardly released any extracellular DNA (Fig 1d and S1 Fig). Representative images of ETs released by PMA stimulated coelomocytes are shown in Fig 1a. The extDNA not only stretched outside of single cells but it appeared to connect several coelomocytes.

In ourstudies, we used Sytox dyes which stain DNA and are commonly used to stain for NETs in mice, e.g. [56,60], fish [28] and invertebrates [29]. The dyes are vital i.e. they stain not only extDNA but also enter the cells with compromised membrane (e.g. necrotic cells) and stain their nuclei [43,44]. Because of this, we verified that after 24 hrs of culture/stimulation majority of the coelomocytes are vital with only some cells taking in the Sytox dyes (e.g. Fig 1b and S2 Fig). Unlike neutrophil nuclei, those of coelomocytes are round, and thus uncharacteristic. For this reason, it is more difficult to distinguish between necrotic cells (with intracellularly stained round nuclei) and those that released ETs and their bodies are surrounded by extDNA (round body-like shaped structures), the latter commonly seen in mice [60]. To clarify which cells are in fact imaged, necrotic or ET-covered/ETting (in a process of releasing ETs) cells, we simultaneously analyzed cell morphology in the brightfield and in green fluorescent channel taking advantage of eleocytes green autofluorescence due to the presence of riboflavin (S2 Fig). Moreover, distinctive morphology of amoebocytes and eleocytes can be observed in Fig 1c. We could observe that majority of coelomocytes that stained with the Sytox dyes were in a process of releasing ETs rather than being dead and passively absorbing the dye. In addition, we excluded that apoptosis or autophagy contributed to the signal (see below). To furthermore verify that majority of coelomocytes are vital we stained the cells simultaneously with acridine orange and Sytox orange (S2b Fig). Acridine orange turns green when it binds to dsDNA thus it stains green all cells that contain nuclei (dead or alive). Of note, earthworm coelomocytes can be divided into two major populations of eleocytes and amoebocytes [1]. The latter cells are not autofluorescent, strongly adhere to plastic/glass surfaces and their cytoplasm is translucent. In general, alive cells absorb (take up) acridine orange (green color) but not Sytox orange (orange/red color). Because of this, the live cells remain green (acridine orange^+^ Sytox orange^-^), while necrotic cells absorb both dyes (acridine orange^+^ Sytox orange^+^) and their nuclei change color to yellow (S2b Fig). Eleocytes are more difficult to differentially stain by the above method due to their autofluorescence. Therefore in the case of autofluorescent green eleocytes which are already green, when their nuclei stain additionally in green (in live cells) by acridine orange, the signal cannot be distinguished from autofluorescence. However, if Sytox orange penetrates membrane of necrotic cells, they turn yellow (S2b Fig).

Interestingly, we captured some of the coelomocytes while they were in a process of ETting and on some occasions we could see a DNA bleb being extruded out of the cells (Fig 1c). Moreover, we detected that both of the populations have capacity to release ETs upon stimulation with PMA (Fig 1c). Moreover, bacteria *X*. *bovienii* (BACT) were effective in induction of ETs by coelomocytes (Fig 1d).We also monitored kinetics of extDNA release by stimulated coelomocytes after their seating *ex vivo* in slide chambers, testing following time points 1, 2, 4, 6, 8 and 24 hours after either PMA or BACT treatment. First signs of released extDNA were observed after 2 hours, but 24 hours were optimal for formation of significant amounts of ETs (data not shown), and therefore this time point was used in subsequent studies.

### ETs are composed of DNA, histones and HSP27

**Fig 2:**
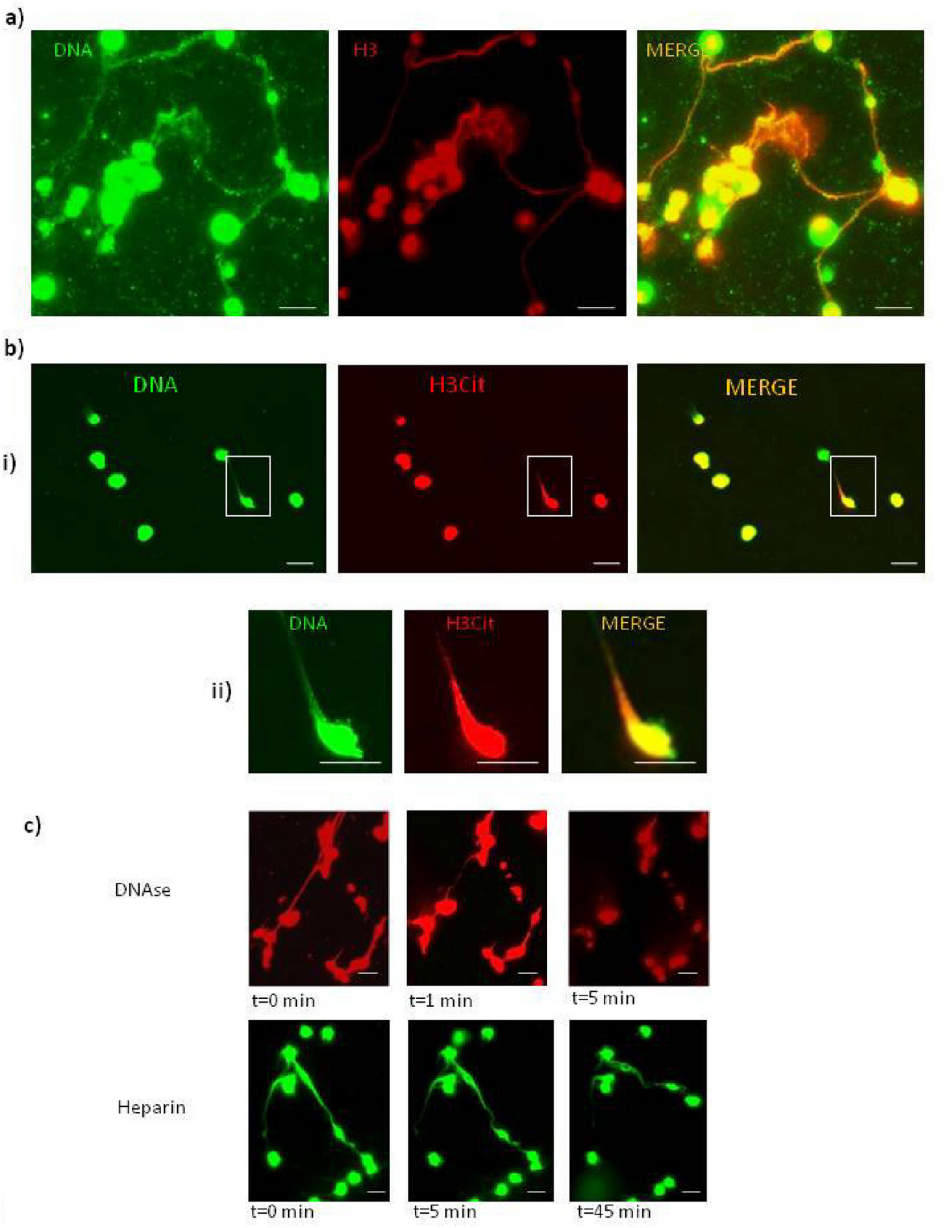
ETs are composed of DNA, citrullinated histones and are DNase- and heparin-sensitive. Representative images of immunofluorescence staining of ETs released by *E*. *andrei* coelomocytes collected from earthworms treated for 24 hrs with bacteria *X*. *bovienii* (BACT, a) or PMA (b, c). Retrieved coelomocytes were seated in slide chambers and the immunostaining was performed after 24 hours; additionally, Sytox orange or Sytox green were used to counterstain extDNA. Immunostaining with specific antibodies revealed that extDNA (green) is decorated with histones 3 (H3, red) (a). Immunostaining with antibody detecting citrullinated histones 3 (H3Cit, red) demonstrated the H3cit signal in co-localization with extDNA (green). i) A larger field of view (FOV) is presented and ii) a higher magnification of the inter-selected FOV. c) disintegration of extDNA released by coelomocytes collected from PMA stimulated earthworms by DNase (red) and heparin treatment (green) shown as time-lapse images. Scale bar on all images 20 μm.

Immunofluorescent staining of the *ex vivo* studied coelomocytes revealed that the released extDNA is decorated with histones (H3; Fig 2a and S3 Fig) and heat shock protein 27 (HSP27; S4 Fig) fulfilling definition of extracellular traps being composed of extDNA and proteins of nuclear/granular/cytoplasmic origin. The histones and HSP27 clearly co-localized with extDNA, and this pattern was similar after stimulation with either PMA or BACT (representative pictures for BACT are shown on Fig 2a and S4 Fig). The networks of ETs stretched between coelomocytes formed remarkable structures that were clearly composed not only of extDNA but also histones H3 (Fig 2a). Images obtained under higher magnification show that histones are distributed along extDNA fibers and furthermore that bacteria bind to such structures (S3 Fig). As shown on S4 Fig (right panel, BACT vs. CTR), in the stimulated cells the expression of HSP27 was detectable in the nucleus region but also along extDNA, whereas in CTR a weak HSP27 signal was observed only in the cytoplasm.

### Histones attached to ETs might be citrullinated

**Fig 3:**
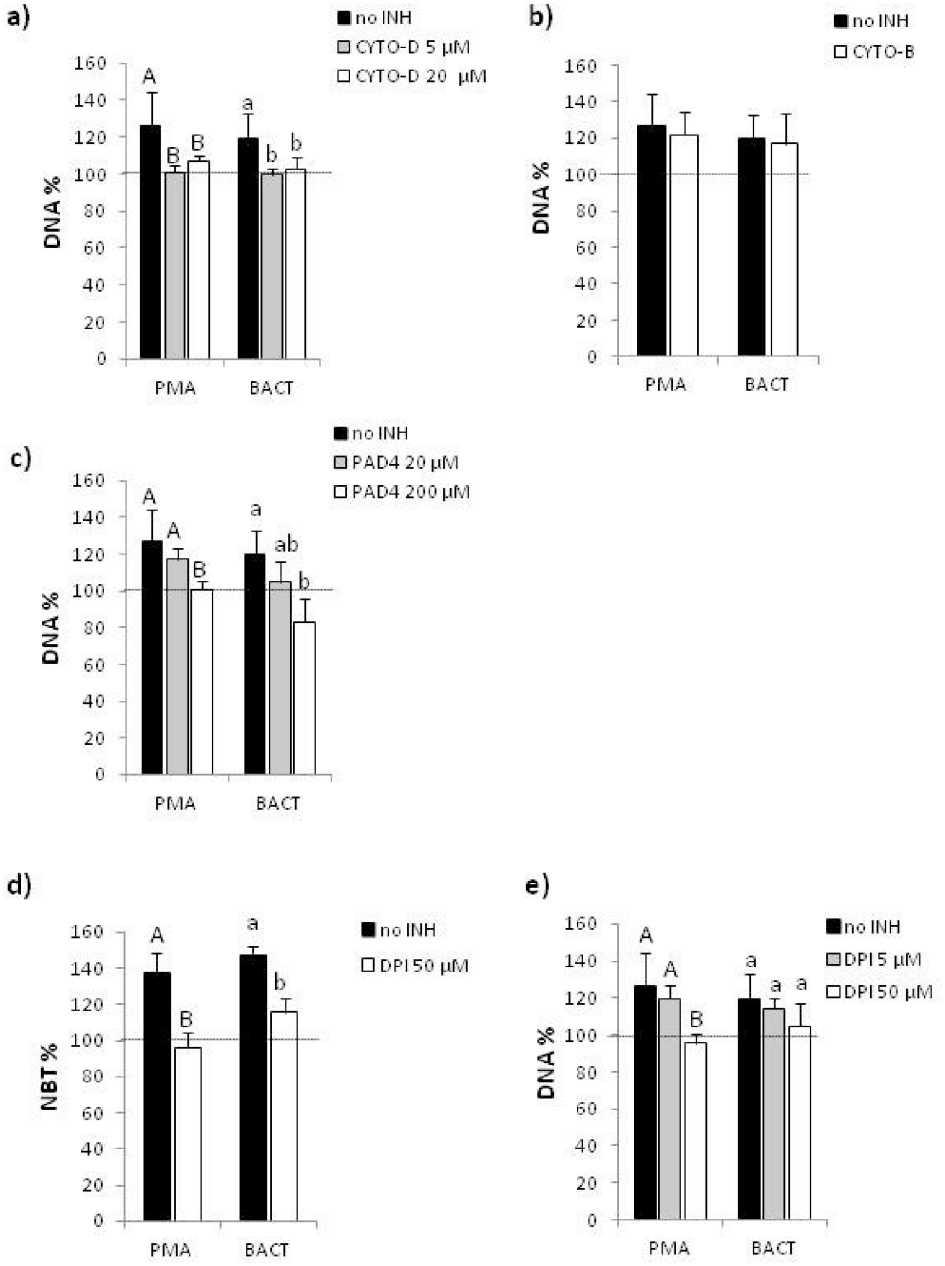
ET formation depends on actin polymerization and partially on NADPH oxidase. Coelomocytes were isolated from *E*. *andrei* and *in vitro* stimulated for 24 hours with either PMA or bacteria *X*. *bovienii* (BACT). Prior to stimulation with PMA/BACT, some cells were pretreated with inhibitors as specifically indicated below. After addition of Sytox orange, the amount of extDNA was evaluated spectrofluorometerically. Effects of a) cytochalasin D inhibitor (CYTO-D), b) cytochalasin B inhibitor (CYTO-B), and c) peptidylarginine deiminase inhibitor (PAD4) on intensity of ETs formation measured by release of extDNA. d)Measurement of respiratory burst (NBT reduction) of coelomocytes isolated from *E*. *andrei* and *in vitro* stimulated for 24 hours with either PMA or BACT.Prior to stimulation with PMA/BACT, some cells were pretreated with DPI, an inhibitor of NADPH oxidase. e) Effects of DPIon intensity of ETs formation measured by release of extDNA. Each experiment was repeated at least 3 times and in each of them 3 to 4 individuals were used. Mean+SD, data for unstimulated cells are expressed as 100% and marked with a horizontal line, different letters (e.g. a vs. b or A vs. B) indicate statistically significant differences between the groups at p<0.05, according to ANOVA. Capital letters (A or B) indicate differences between coelomocytes activated with PMA but pre-treated with different inhibitors, while small letters (a or b) indicate differences between coelomocytes activated with bacteria (BACT) but pre-treated with different inhibitors.

Immunostaning of coelomocyte cultures (the *ex vivo* setting) revealed that histones H3 which are attached to ETs might be citrullined as they reacted with the anti-H3 antibody (H3cit, representative images for PMA stimulated cells, Fig 2b). As the process of histone citrullination depends on actin polymerization, we applied cytochalasin D (Cyto-D) to suppress this process in the *in vitro* setting. Cyt-D inhibited ET formation in either of applied doses by app. 20% when PMA was used to induce ETs and 14–17% for bacteria-induced ETs (Fig 3a). In contrast, another actin polymerization inhibitor, cytochalasin B (Cyto-B) which strongly inhibits phagocytosis by coelomocytes, applied in the same manner as Cyto-D, did not (Fig 3b).We also tested the effect of PAD4 inhibitor on ET formation. The dose of 200 μM, unlike 20 μM, significantly inhibited release of extDNA (Fig 3c).

### ETs are DNase- and heparin-sensitive

We tested sensitivity of extDNA released by coelomocytes injected with PMA (Fig 2c) and BACT (not shown) to DNase and heparin (the *ex vivo* setting). Extracellular DNA released by the cells was almost completely dissolved after 5-minute stimulation with DNase I (representative time-lapse images of PMA-induced ETs, Fig 2c top). Also heparin disintegrated ETs but after much longer incubation of 45 minutes (representative time-lapse images of PMA-induced ETs, Fig 2c, bottom).

### Bacteria-induced ETs, unlike PMA-induced, are NADPH oxidase-independent

Isolated coelomocytes were *in vitro* treated with either PMA or BACT for 24 hrs and both agents triggered respiratory burst which was inhibited by diphenyleneiodonium (DPI) by 40% and 30%, respectively (Fig 3d). DPI is an inhibitor of NADPH oxidase and thus an agent limiting generation of ROS [39]. Despite this fact, DPI inhibited ET release only when applied to coelomocytes prior to PMA (by app. 30%), but not BACT stimulation (Fig 3e), indicating that *X*. *bovienii*-induced extracellular traps are formed independently of ROS.

### Formation of ETs is independent of coelomocyte apoptosis or autophagy

To block apoptosis we applied a multi-caspase activity inhibitor VAD (S5a Fig) and a more specific inhibitor for caspase 3 (data no shown). To check autophagy we used wortmannin and all of the studies were performed in the *in vitro* setting. Neither of the inhibitors affected ET formation (S5b Fig).

### ET formation by coelomocytes depends on protease activity

**Fig 4:**
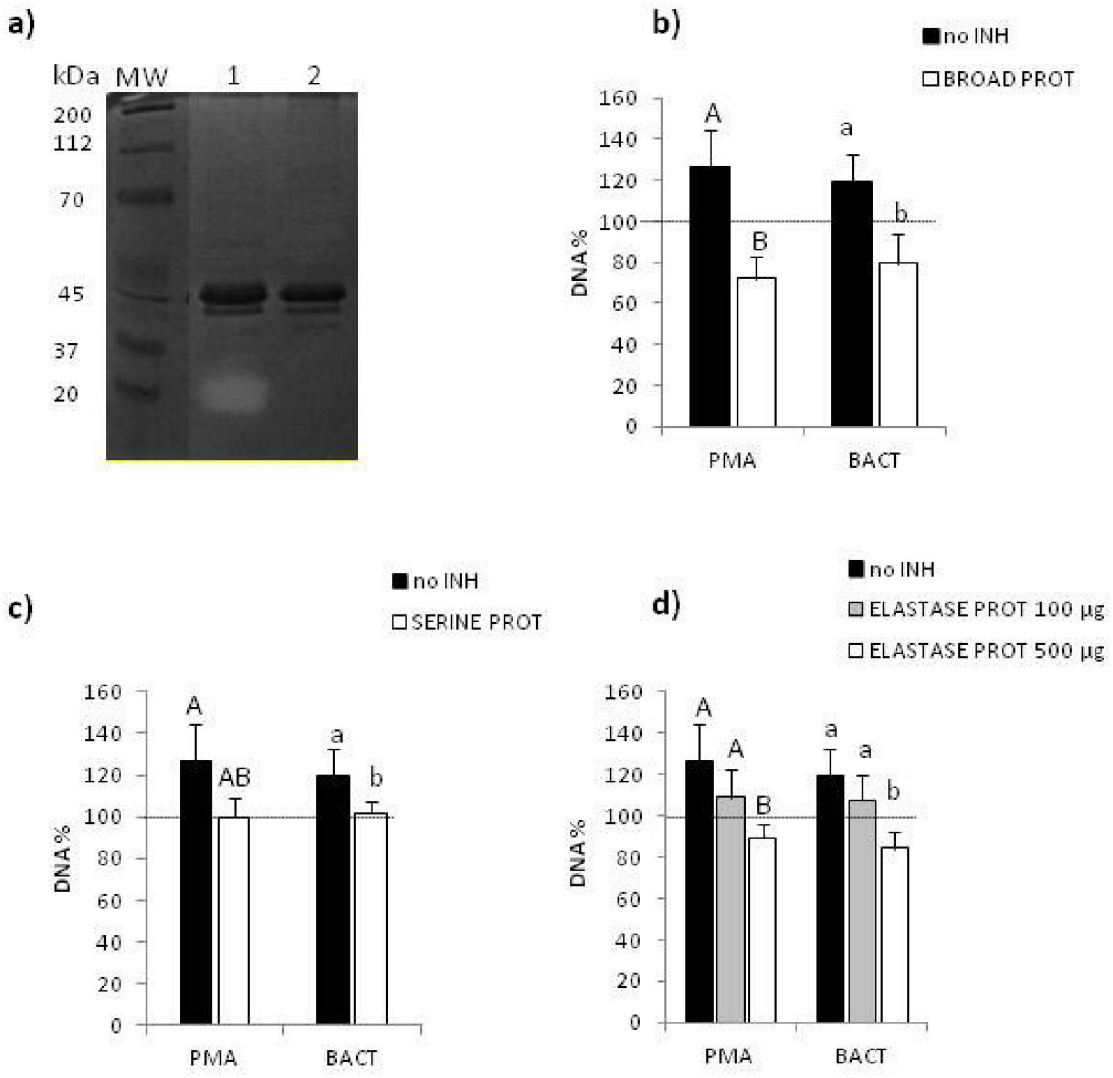
Serine protease activity is required for ET formation by coelomocytes. a) A representative zymographic gel of proteolytic activity (clear bands) detected incoelomic extract of *E*. *andrei*obtained after 24-hour*in vivo* stimulation with PMA (lane 1). Some animals were pretreated before PMA injection with a broad protease inhibitor (BROAD PROT) (lane 2). b-d) Coelomocytes were isolated from *E*. *andrei* and *in vitro* stimulated for 24 hours with either PMA or bacteria *X*. *bovienii* (BACT). Prior to stimulation with PMA/BACT, some cells were pretreated with inhibitors as specifically indicated below. After addition of Sytox orange the amount of extDNA was evaluated spectrofluorometerically. Effects of b) BROAD PROT, c) a serine protease inhibitor (SERINE PROT), and d) neutrophil elastase inhibitor (ELASTASE PROT) on intensity of ET formation measured by release of extDNA. Each experiment was repeated at least 3 times and in each of them 3 to 4 individuals were used. Mean+SD, data for unstimulated cells are expressed as 100% and marked with a horizontal line, different letters (e.g. a vs. b or A vs. B) indicate statistically significant differences between the groups at p<0.05, according to ANOVA. Capital letters (A or B) indicate differences between coelomocytes activated with PMA but pre-treated with different inhibitors, while small letters (a or b) indicate differences between coelomocytes activated with bacteria (BACT) but pre-treated with different inhibitors.

The broad-spectrum inhibitor of proteases (BROAD PROT) was injected into earthworms prior to their stimulation with PMA (the *in vivo* setting). Subsequently, their coelomic fluid was isolated and analyzed by zymography, a method which allows detection of such proteases as gelatinases, collagenases and matrilysin. The inhibitor completely inhibited any proteolytic activity which can be detected by this method i.e. two proteolytic (clear) bands present in the coelomic extract of PMA-stimulated earthworms were not detectable when animals were pretreated with BROAD PROT (Fig 4a). Subsequently, coelomocytes isolated from unstimulated earthworms were pretreated with BROAD PROT prior to PMA or BACT treatments (the *in vitro* setting) and the inhibitors significantly diminished ET formation (Fig 4b). To narrow down which group of proteases is mainly involved in ET formation, we used another protease inhibitor in the same system. Pretreatment with this inhibitor (named here SERINE PROT) also significantly inhibited ET formation (Fig 4c). Finally we used a selective inhibitor of neutrophil elastase (ELASTASE PROT) shown to inhibit elastase-like activity in different vertebrates [61], as elastase-like activity does operate also in earthworms [36]. Both applied doses of ELASTASE PROT significantly down-regulated ET release (Fig 4d).

### Earthworms cast ETs also *in vivo* in their coelom

**Fig 5:**
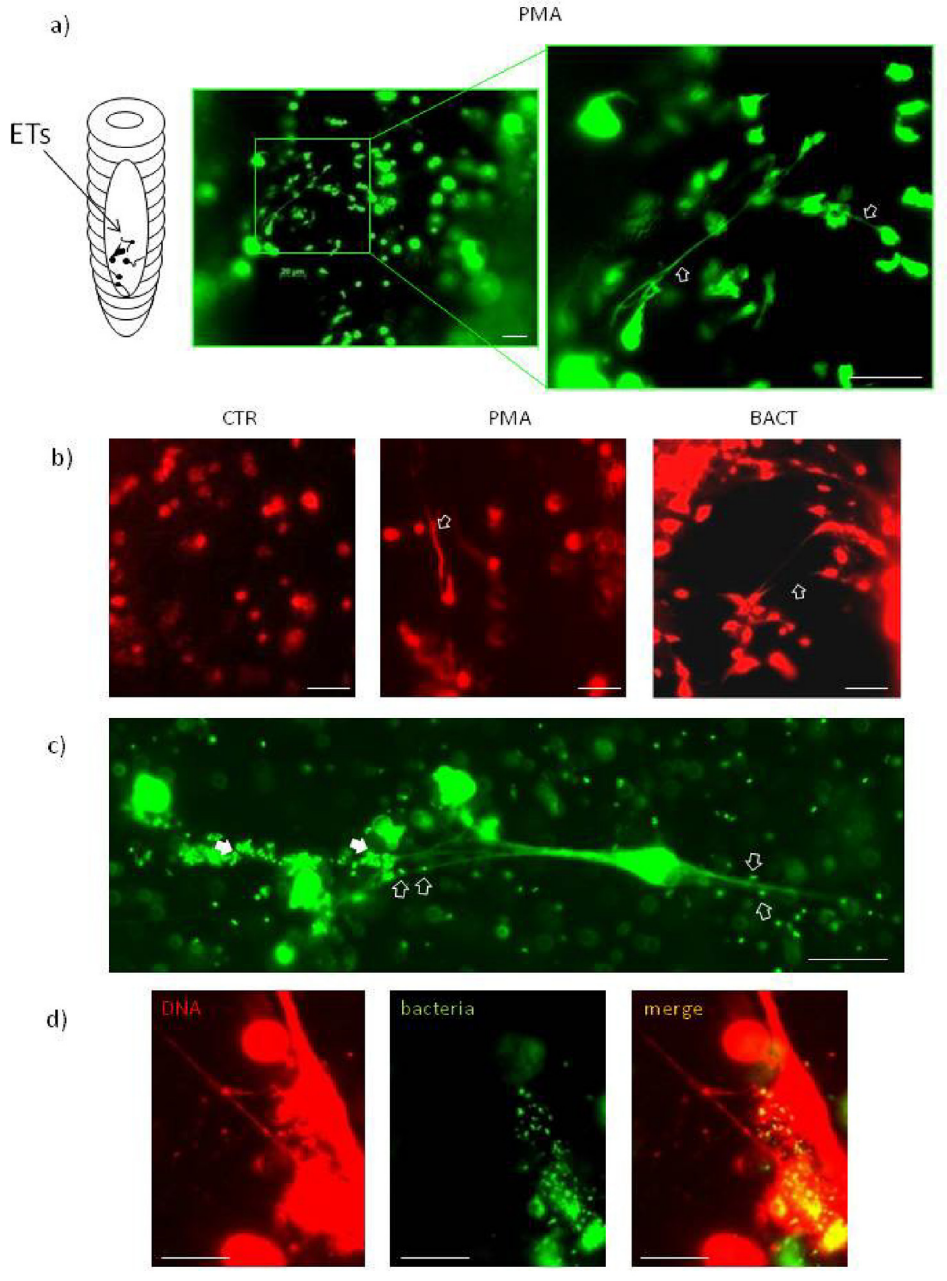
ETs are formed *in vivo* in the coelomic cavity of earthworms and they trap bacteria. Earthworms were injected with either PMA or bacteria *X*. *bovienii* (BACT) for 24 hrs, then their skin and the coelomic cavity were cut open and the content of the cavity was observed under a fluorescent microscope. a) (Left) schematic illustration of an earthworm body with exposed coelomic cavity and marked areas where coelomocytes and ETs were imaged. (Right) Representative images of ETs released in response to *in vivo* injection of PMA 24 hrs prior to imaging. ETs were stained with Sytox orange (shown in false green color). b) presence of ETs in the coelomic cavity of control untreated earthworms (CTR) and animals treated for 24 hrs with either PMA or BACT. c) Trapping of bacteria: ETs formed by coelomocytes isolated from BACT-treated *E*. *andrei* (for 24 hrs), then were seated in slide chambers and stained with Sytox orange (shown in false green color) after 24 hrs. d) Trapping of stained bacteria: in some studies earthworms were injected with Sytox green pre-stained BACT (otherwise protocol as described in b). After 24 hrs in slide chambers, ETs were counterstained with Sytox orange. Scale bar on all images 20 μm.

ET formation was also confirmed in situ, directly in the *E*. *andrei* coelom (Fig 5a). Fibers of extDNA were observed to connect coelomocytes, similarly as seen in the *ex vivo* conditions in slide chambers (Figs 5b vs. 1a and 1b).

### Earthworm ETs trap bacteria

In the *ex vivo* setting we imaged bacteria *X*. *bovienii* trapped by ETs (Fig 5c). The captured bacteria could be seen as single cells, or whole aggregates of bacteria were trapped by ETs (Fig 5c and 5d). As bacteria were capturing Sytox staining themselves, in some experiments we counterstained them *in vitro* with another Sytox dye (e.g. bacteria were stained with Sytox green prior to *in vivo* injection while at the time of imaging we stained extDNA with Sytox orange). In this setting, green bacteria captured by red extDNA are seen as yellow (Fig 5d).

### Coelomocytes releasing ETs form aggregates

**Fig 6:**
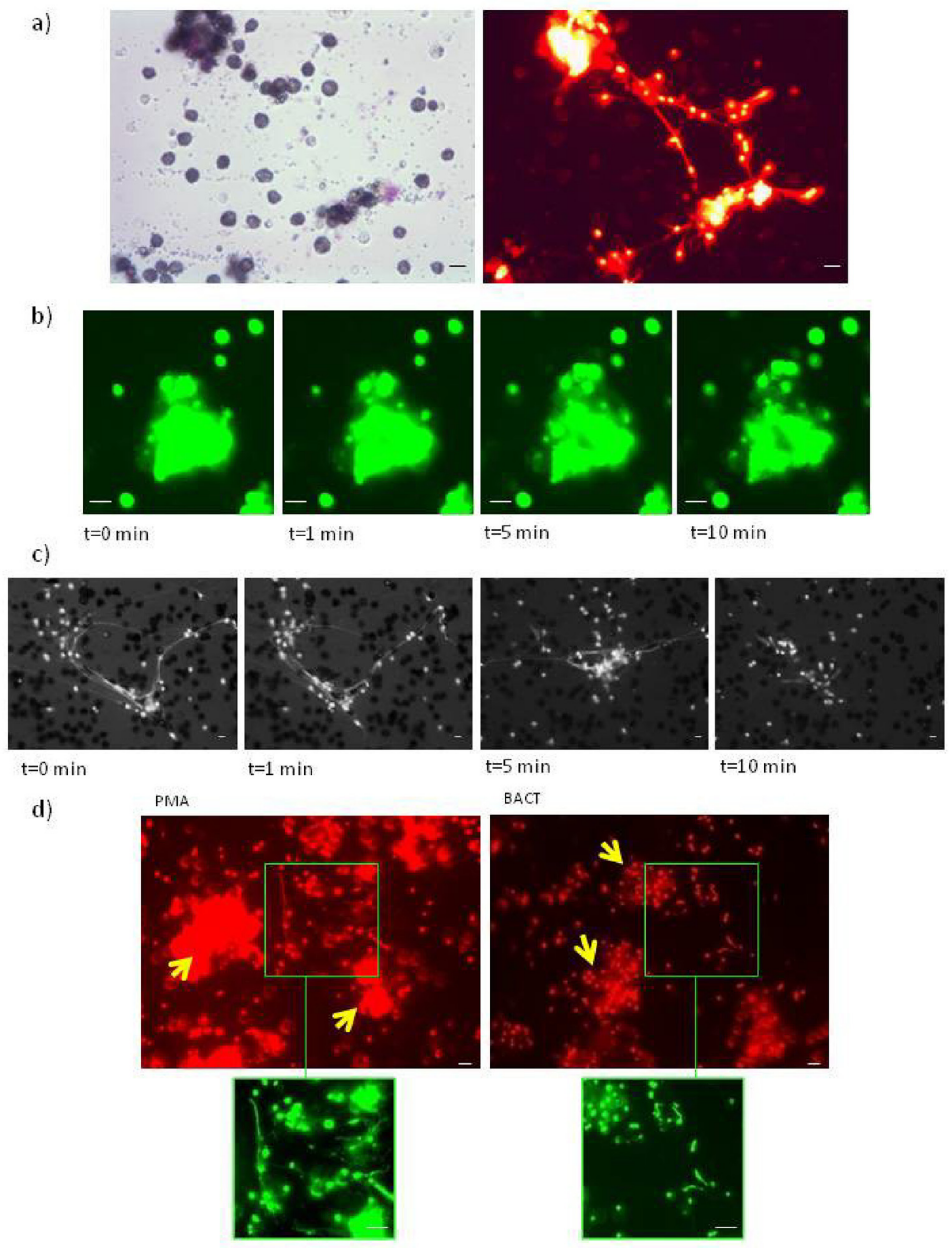
Coelomocytes forming ETs aggregate and extracellular DNA covers capsules entrapping bacteria. a-c) Coelomocytes were isolated from *E*. *andrei* and *in vitro* stimulated for 24 hours with either PMA or bacteria *X*. *bovienii* (BACT). a) Sytox orange (red) staining of extDNA revealed that aggregates of coelomocytes are interconnected by ETs but also the cell aggregates are covered with extDNA. Representative images show data for BACT stimulated coelomocytes; bright field was used to show localization of coelomocytes (left) and fluorescence to reveal localization of extDNA (right). b) Representative time-lapse images of coelomocytes aggregates are covered by extDNA (false green). DNase-treated aggregates c) smaller aggregates of coelomocytes loose connection with each other after removal of extDNA. Representative sensitive time-lapse images of the aggregates after DNAse treatment (simultaneously bright filed is show for visualization of coelomocytes and fluorescence to visualize ext DNA). d) formation of ET-interconnected coelomocyte aggregates was also confirmed *in vivo*: coelomocyte aggregates formed upon earthworm injection with either PMA or BACT were imaged 24 hrs post stimulation. ExtDNA was stained with Sytox orange (red; shown in false green colour on enlarged fragments of images). Scale bar on all images 20 μm.

In our *ex vivo* studies (Fig 1b), we observed that coelomocytes forming ETs tend to form aggregates, and/or formation of ETs initiates this process. In further *ex vivo* experiments, we observed that these aggregates were forming large structures, and the aggregates were connected by extDNA of ETs (Fig 6a).When we added DNase we observed that the structure of the aggregates loosens up and some border coelomocytes are detaching from the structure (Fig 6b, representative time-lapse images of PMA-induced ETs). Also, addition of DNase broke connections between smaller aggregates (Fig 6c, representative time-lapse images of PMA-induced ETs). Formation of similar coelomocyte aggregates interconnected by extDNA/ETs was confirmed *in vivo* (Fig 6d).

## Discussion

Earthworms are characterized by presence of the coelom and the closed vascular system which were central for emergence of some novel and effective immune mechanisms which operate in these animals [62]. This is especially important considering that the coelomic cavity of earthworms is not aseptic and always contains bacteria, protozoans and fungi as the cavity is opened to the outer milieu by dorsal pores [1]. Thus one of the mechanisms to keep the pathogens at bay is presence of numerous coelomocytes (1 pathogen:10 phagocytic cells) floating in the coelomic fluid which are capable of performing immunological functions such as phagocytosis, cell lysis and encapsulation [1].

In our previous studies, we verified effectiveness of cellular and humoral defense mechanisms operating in *E*. *andrei* in response to numerous immunostimulants such as LPS, *Micrococcus luteus*, and PMA and we reported that the two systems are highly activated in response to these factors [15,63]. In particular, we showed that activity of prophenoloxidase (pro-PO) system (the critical system of invertebrate humoral immune response) is activated by the above stimulants as well as number, composition, proliferation and viability (increased apoptosis) of coelomocytes is changed [15,63]. Moreover, respiratory burst forming reactive oxygen species (ROS) is highly activated.

Here we extend the list of earthworm defense mechanisms by demonstration that coelomocytes can also cast extracellular traps (ETs) which successfully trap bacteria. Catching of bacteria was also observed in the case of shrimp ETs released by *M*. *japonicus* [31] and *L*. *vannamei* [29,30], and the structures were further shown to kill *Escherichia coli* [29,30]. Here we observed formation of ETs in multiple settings i.e. by *in vitro* stimulated isolated coelomocytes, *ex vivo* (when coelomocytes were isolated from *in vivo* treated earthworms but released ETs in culture conditions) and most importantly *in vivo*, directly in the coelomic cavity. And ETs were formed in response to various pathogens (Gram^-^ and Gram^+^ bacteria) or their derivatives (LPS, fungi polysaccharide), and ROS (H_2_O_2_), in addition to a very well established ET-inducer, PMA [17,22,38]. Under all these conditions, DNA was observed to be extruded by coelomocytes. The released DNA usually stayed attached to the cells, in some cases interconnecting them, but we also captured ETs emanating from a coelomocyte surface. Interestingly, we observed that both immunocompetent coelomocyte populations, eleocytes and amoebocytes, release ETs and in fact in mammals not only neutrophils but also macrophages and eosinophils were demonstrated to form the extracellular traps [18,26,27]. To confirm that Sytox-positive fibers are indeed made up by DNA present outside of coelomocyte bodies, we added DNase and the enzyme completely removed extDNA within minutes. As extDNA can be released from cells simply after their necrotic death, we verified if histones are also attached to extDNA as this is one of the highlights of ETs/NETs [16]. Indeed, we clearly observed that histone signal can be detected along the whole length of extDNA. One of the challenges of working with non-vertebrate animals is a limited number of tools and in particular antibodies which can be used. On the other hand, histones are among the most conserved proteins in eukaryotes, including earthworms [62], and vertebrate antibodies were successfully used to identify earthworm histones [62]. Moreover, antibodies detecting modified histones, e.g. their phosphorylated form, were also specific in earthworms [62]. To further secure specificity, to detect histones we used an antibody which was previously shown to detect H3 in *Drosophila melanogaster* [64]. The antibody-detected attachment of histones to extDNA was also observed in two other studies on invertebrate ETs: in the shrimp [29] and shore crab [33]. However, we undertook one more approach to confirm attachment of histones to ETs by showing that overtime ETs are destabilized by heparin. Attachment of histones to extDNA was also showed in some other invertebrate species. Namely, H1 in shrimps and oyster [29,32], H2A in crab [33] and H5 in oyster [32] were reported to decorate ETs.

Moreover, not only did we show that H3s are attached to ETs but in fact we reveal that they might be citrullinated. To test it, we applied an antibody (from the same supplier as for H3) which detects only citrullinated H3 in positions R2/R8/R17, and showed its attachment to ETs. Presence of citrullinated histones attached to ETs/NETs is another highlight of the structures in vertebrates [45], and PAD4 is the enzyme which deiminates multiple arginine sites on histones H3 (R2, R8, R17, and R26) [65]. However, there are no available data supporting existence of PAD4 in taxa lower than Cephalochordates, except of bacteria *P*. *gingivalis* [37], and in fact in invertebrates the process of citrullination has not been studied yet. Nevertheless, we did apply PAD4 inhibitor (Cl-amidine) in our *ex vivo* and *in vivo* experiments and observed significant limitation of ET release. As in neutrophils PAD4 activation requires actin polymerization which can be inhibited by cytochalasin D [25,33], we applied this inhibitor in our studies and it limited ET formation, both by isolated coelomocytes and *in vivo* in the coelom. However, the mechanism by which H3 could become citrullinated in our system cannot be admittedly explained with the current state of knowledge.

NET formation, especially the PMA-dependent, was shown to be frequently associated with increased respiratory burst and ROS formation [22]. Here we confirmed that also earthworm PMA-induced ETs are ROS depended, however *X*. *bovienii*- stimulated ETs did not require ROS. The latter phenomenon, when more physiological ET inducers (often bacteria) lead to ROS-independent ET formation, was also observed in vertebrate systems [3,28,56]. NADPH oxidase-dependent ET formation upon PMA stimulation was also observed in other invertebrates, e.g. crab *C*. *maenas* [33]. However, in social amoeba *Dictyostelium discoideum* also LPS-induced ETs were ROS-dependent [34] thus this phenomenon seems to be species-specific.

ET/NET formation is believed to occur when phagocytes cannot cope with exceptionally numerous pathogens or the invaders are too big to be phagocytized [16,18]. But in such situation, overwhelmed phagocytes might also die by either apoptosis or autophagy [66]. However, we excluded involvement of these processes in formation of ETs by coelomocytes by application of appropriate inhibitors.

We also identified that heat shock protein 27 (HSP27) is attached to *E*. *andrei* ETs. HSPs are chaperon proteins that are abundantly expressed by stressed coelomocytes, not only in response to thermal injury but also upon LPS or PMA stimulation [15]. Attachment of another HSP (HSP72) to NETs was shown previously when human neutrophils were stimulated with *Mycobacterium tuberculosis* [67]. However, HSP72 was absent in PMA-induced NETs which led to conclusion that HSP72 are sequestered to NETs once they are released by the cells. In our model, HSP27 was attached to both *X*. *bovienii* and PMA-induced ETs suggesting that most probably it is attached to ETs already upon their formation. In fact, we chose to detect HSP27 out of other HSP molecules as it enters nuclei only in stressed cells [68]. Accordingly, in control coelomocytes HSP27 signal was only weakly present in the cytoplasm while clearly detectable in the nucleic area, or attached to ETs, upon *X*. *bovienii* and PMA stimulation.

Along with PAD4, neutrophil elastase (NE) was shown to actively participate in NET formation as it degrades histones [22]. In vertebrates, expression of NE is most abundant in neutrophils, although it is not limited to these cells [69]. Elastase-like activity is also present in earthworms [70,71]. NE and earthworm elastase-like proteases belong to serine proteases which are abundant and dominant in earthworms and can degrade not only elastin, but also gelatin, casein, and possess fibrinolytic activity also towards vertebrate proteins e.g. porcine elastase, human plasminogen, for review see [72]. The molecular weights of earthworm proteases are in a range of 20–35 kDa [72] and we detected their presence within this range with the gelatin zymography. Broad proteases inhibitor completely inhibited this activity and when we used it either *in vitro* or *in vivo*, it did significantly inhibit ET formation. To more specifically identify proteases which might participate in ET release we also tested a serine protease inhibitor showed previously to successfully inhibit these earthworm enzymes [56]. Also this inhibitor significantly affected ET release confirming that these are serine proteases which are required for ET release by coelomocytes. Finally, we tested the inhibitor of human NE and we observed a similar effect. As structure and active site of human NE and earthworm elastase-like enzymes were never compared, it is cautious to conclude that these are serine proteases, including elastase-like activity, that are required for ET formation in earthworms.

During our studies of ET formation, both in *ex vivo* and *in vivo* conditions, we observed that their release and the process of formation of pigmented capsules might be connected and/or complement each other. In particular, we detected that ETs not only stretched outside of coelomocytes but also connected adjacent cells, and furthermore, coelomocytes releasing ETs often aggregated and the aggregates were covered with extDNA. Additionally, also the aggregates were connected to each other by extDNA. Aggregation of coelomocytes proceeds a process of encapsulation i.e. formation of pigmented multicellular nodules (brown bodies) which neutralize foreign material by surrounding them [1]. Encapsulation allows for removal of foreign bodies (brown bodies are eventually expelled from the earthworm by autotomy) but melanin covering the capsules also has bacteriostatic properties [73]. Formation of melanin occurs in coelomic fluid and in a subpopulation of granulocytes [74]. On the other hand, amyloid fibrils production is due to exocytosis of circulating cells (amoebocytes/granulocytes). In invertebrates, amyloid fibrils adhere to the non-self molecules/organisms driving the pigment accumulation close to the invaders, avoiding the toxic melanin dispersion in hemocelic environment [74–77].

Importantly, amyloid fibrils also accumulate in mammals, thus are evolutionary conserved [74], but their deposition mostly leads to pathological diseases such as Alzheimer disease [78,79]. Interestingly, amyloid fibrils can induce formation of ETs by neutrophils [80] but they also attach to neutrophil ETs where they address raptured proteins and DNA against the non-self [80].

Formation of melanin/encapsulation is activated by a cascade of serine proteases [74]. In the current study, we observed the first stages of encapsulation, aggregation of the cells. Therefore we hypothesized that once activated serine proteases might facilitate simultaneously both processes, and ETs help to keep coelomocytes together during formation of the aggregates as ETs are electrostatically-charged adhesive networks [16,45]. To confirm it, we treated the aggregates with DNase and observed that their structure becomes less coherent over time when extDNA is not present to keep the structure together. Moreover, we observed in the *ex vivo* studies that single coelomocytes releasing ETs stay attached to the surface while aggregates float freely, only connected to each other by fibers of ETs. Therefore, we would hypothesize that ETs help smaller coelomocytes meshworks form bigger aggregates that would eventually be enclosed in a capsule. In fact, we were able to show that DNase disrupted connections between smaller aggregates of coelomocytes, possibly preventing development of even bigger structures. Aggregates of ET forming haemocytes were also showed in shore crab *C*. *maenas*, and chromatin and a crustacean homologue of MPO were shown to hold the structure together [33].

In conclusion, we show that also earthworms cast extracellular traps in response to numerous pathogenic agents and they efficiently trap bacteria. These ETs are composed of extracellular DNA and histones which might be citrullinated but the latter observation requires further studies. The process of ET formation, similarly to that of vertebrates, depends on serine proteases and possibly elastase-like activity. Additionally, formation of ETs seams to facilitate a process of encapsulation, providing previously unrecognized defense mechanisms to the repertoire of Annelida’s immune responses.

## Supporting Information

S1 Fig Intensity of ET formation measured as release of extDNA by immunostimulated coelomocytes. Coelomocytes were collected from either earthworms injected with sodium chloride (CTR) or *E*. *andrei* treated for 24 hrs with PMA (0.1 μg/ml), *X*. *bovienii* (BACT, 5x10^6^/ml), LPS (1mg/ml), *M*. *luteus* (Ml, 1mg/ml), zymosan (Z, 1mg/ml) or H_2_O_2_ (100 mM). Each experiment was repeated at least 3 times and in each of them 3 to 4 individuals were used. Mean+SD, different letters (e.g. A vs. B) indicate statistically significant differences between the groups at p<0.05, according to ANOVA.
(TIF)

S2 Fig Representative images of coelomocytes stimulated with PMA for 24 hrs revealing all poosible cell responses: release of ETs, necrosis and viable cells. a) representative rate/distribution of coelomocytes that release ETs, i) bright field (to visualize cell bodies, ii) bright field overlaid with Sytox orange fluorescent signal (to visalize ETs and ETting cells). b) Co-staining of coelomocytes with extDNA/ETs dectecting dye (Sytox orange) and with dye detecting dsDNA/nucleated cells (acridine orange). Autofluorescent eleocytes (AF*, green fluorecscence is derived from riboflavin) and amoebocytes (^), some coelomocytes are in a process of extruding their DNA (ET) or are necrotic (N); most of cells are only green (due to autofluorescence or acridine orange staining) indicating that they are viable (V) as they do not take in Sytox orange thus do not turn yellow. VAF*–viable autofluorescet eleocytes, V^–viable amoebocytes, N—necrotic cells, ET—extDNA or cells in a proces of releasing ETs. Scale bar of all images 20 μm.
(TIF)

S3 Fig ETs are composed of DNA and histones and trap bacteria. Representative images of immunofluorescence staining of ETs released by *E*. *andrei* coelomocytes collected from control unstimulated (CTR) earthworms or treated for 24 hrs with bacteria *X*. *bovienii* (BACT). Retrieved coelomocytes were seated in slide chambers and the immunostaining with anti-H3 antibody (green) was performed after 24 hrs; additionally, Sytox orange (red)was used to counterstain extDNA. Bacteria (red, stained with Sytox orange) are seen binding to ETs composed of extDNA and H3 (merge images), yellow arrows. White arrows indicate extDNA. Scale bar on all images 20 μm.
(TIF)

S4 Fig ETs are composed of DNA and Hsp27 and trap bacteria. Representative images of immunofluorescence staining of ETs released by *E*. *andrei* coelomocytes collected from earthworms treated for 24 hrs with bacteria *X*. *bovienii* (BACT). Retrieved coelomocytes were seated in slide chambers and the immunostaining with anti-HSP27 antibody (green) was performed after 24 hrs; additionally, Sytox orange (red) was used to counterstain extDNA. Images presented in the right panel also visualize bacteria (red, stained with Sytox orange) binding to ETs composed of extDNA and HSP27 (merge images). Scale bar on all images 20 μm.
(TIF)

S5 Fig Formation of ETs is independent of coelomocyte apoptosis or autophagy. a-b) Coelomocytes were isolated from untreated *E*. *andrei* and *in vitro* stimulated for 24 hrs with either PMA or bacteria *X*. *bovienii* (BACT). Prior to stimulation with PMA/BACT, some cells were pretreated with inhibitors as specifically indicated below. After addition of Sytox orange, the amount of extDNA was evaluated spectrofluorometerically. Effects of a) VAD, pan-caspase inhibitor and b) wortmanin, an inhibitor of autophagy, on intensity of ET formation measured by release of extDNA. Each experiment was repeated at least 3 times and in each of them 3 to 4 individuals were used. Mean+SD, data for unstimulated cells are expressed as 100% and marked with a horizontal line.
(TIF)
