# Supplementary figures and images for: Conservative Mechanisms of Extracellular Trap Formation by Annelida Eisenia andrei: Serine Protease Activity Requirement
Source: PLoS One. 2016 Jul 14;11(7):e0159031. doi: 10.1371/journal.pone.0159031 (PMC4945018; doi:10.1371/journal.pone.0159031)

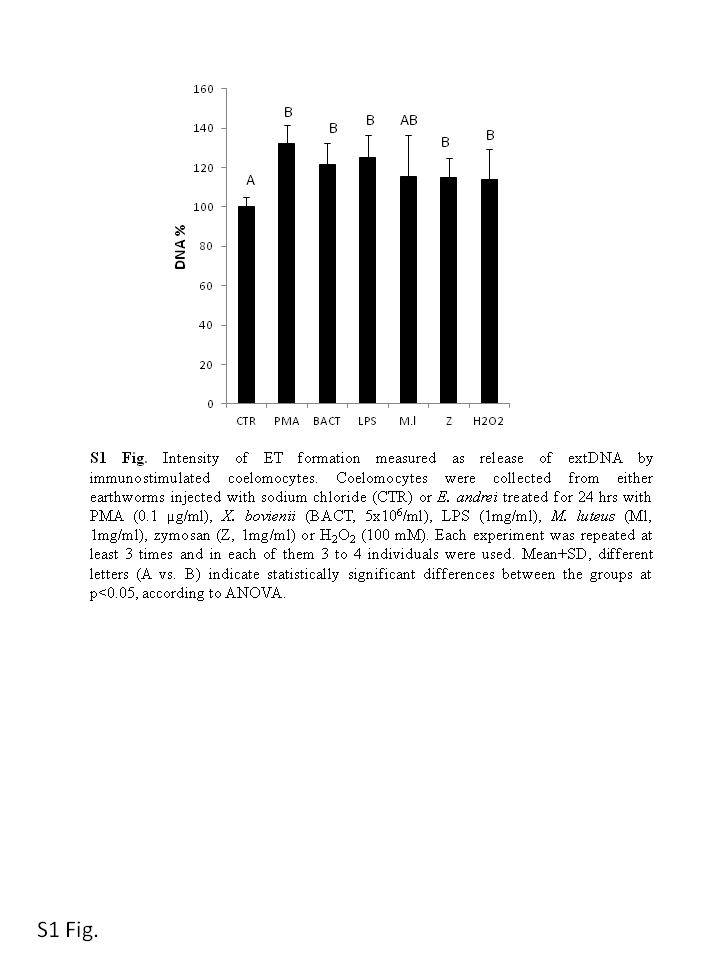

Supplement: S1 Fig — Coelomocytes were collected from either earthworms injected with sodium chloride (CTR) or E. andrei treated for 24 hrs with PMA (0.1 μg/ml), X. bovienii (BACT, 5x10^6^/ml), LPS (1mg/ml), M. luteus (Ml, 1mg/ml), zymosan (Z, 1mg/ml) or H2O2 (100 mM). Each experiment was repeated at least 3 times and in each of them 3 to 4 individuals were used. Mean+SD, different letters (e.g. A vs. B) indicate statistically significant differences between the groups at p<0.05, according to ANOVA. (TIF) [file pone.0159031.s001.TIF]

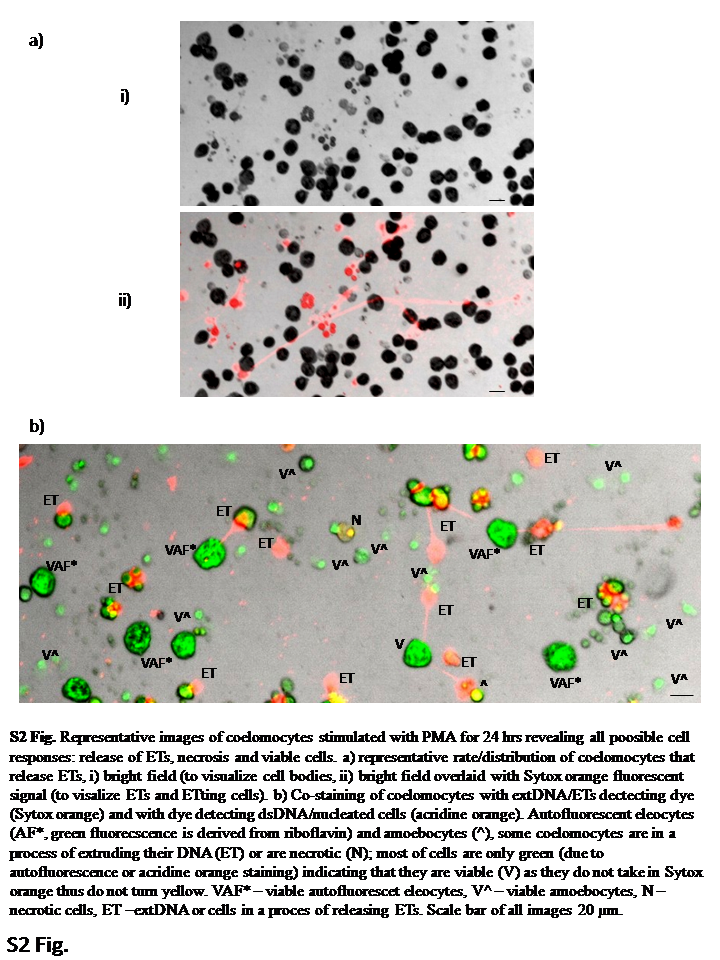

Supplement: S2 Fig — a) representative rate/distribution of coelomocytes that release ETs, i) bright field (to visualize cell bodies, ii) bright field overlaid with Sytox orange fluorescent signal (to visalize ETs and ETting cells). b) Co-staining of coelomocytes with extDNA/ETs dectecting dye (Sytox orange) and with dye detecting dsDNA/nucleated cells (acridine orange). Autofluorescent eleocytes (AF*, green fluorecscence is derived from riboflavin) and amoebocytes (^), some coelomocytes are in a process of extruding their DNA (ET) or are necrotic (N); most of cells are only green (due to autofluorescence or acridine orange staining) indicating that they are viable (V) as they do not take in Sytox orange thus do not turn yellow. VAF*–viable autofluorescet eleocytes, V^–viable amoebocytes, N—necrotic cells, ET—extDNA or cells in a proces of releasing ETs. Scale bar of all images 20 μm. (TIF) [file pone.0159031.s002.TIF]

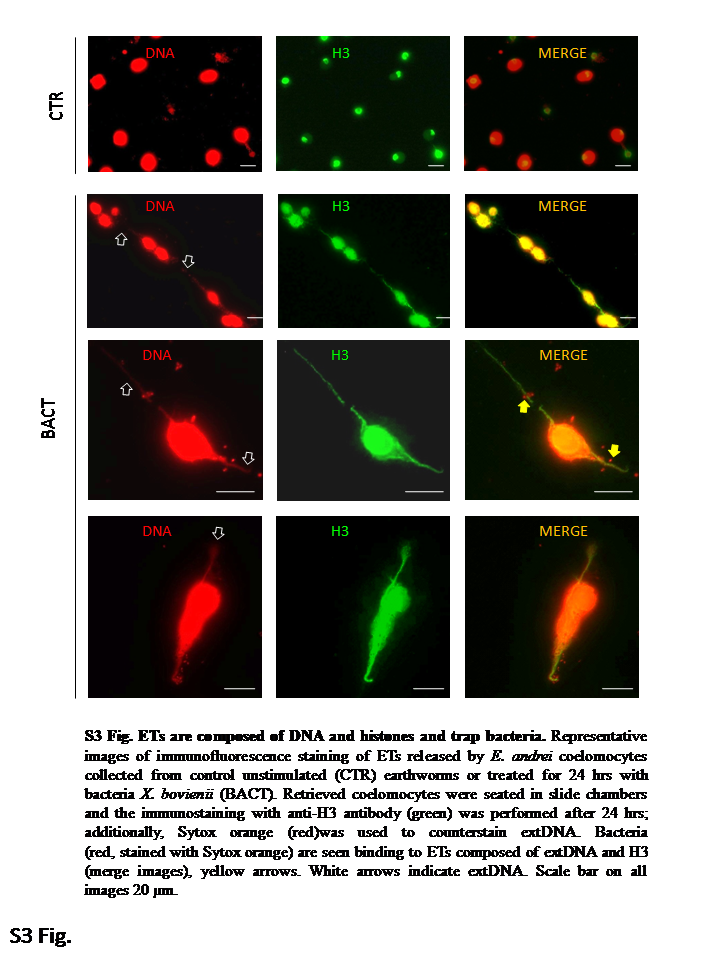

Supplement: S3 Fig — Representative images of immunofluorescence staining of ETs released by E. andrei coelomocytes collected from control unstimulated (CTR) earthworms or treated for 24 hrs with bacteria X. bovienii (BACT). Retrieved coelomocytes were seated in slide chambers and the immunostaining with anti-H3 antibody (green) was performed after 24 hrs; additionally, Sytox orange (red)was used to counterstain extDNA. Bacteria (red, stained with Sytox orange) are seen binding to ETs composed of extDNA and H3 (merge images), yellow arrows. White arrows indicate extDNA. Scale bar on all images 20 μm. (TIF) [file pone.0159031.s003.TIF]

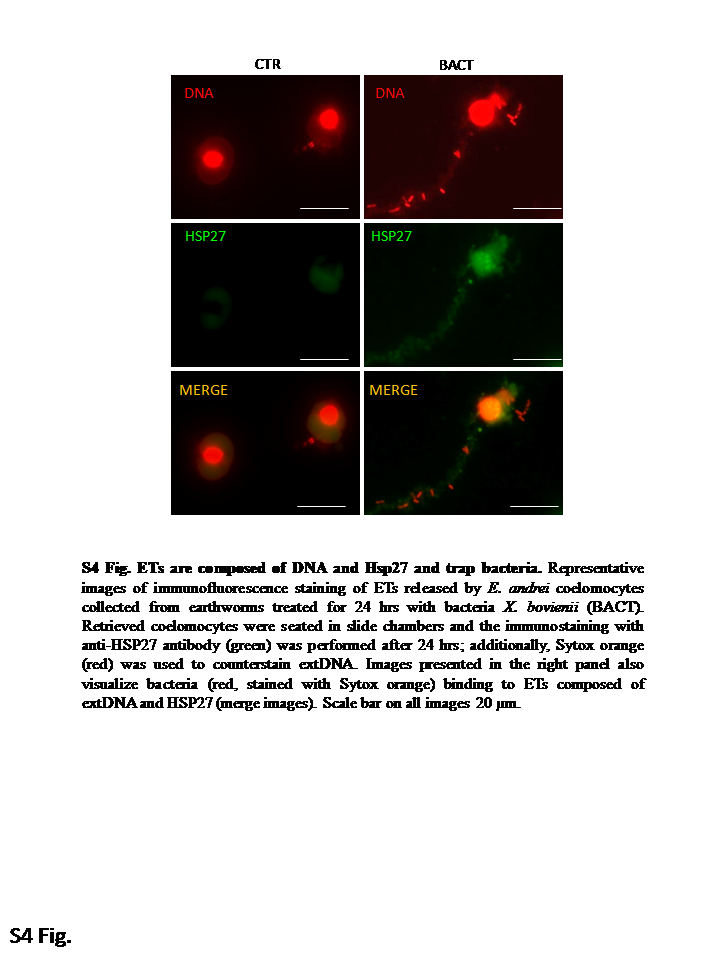

Supplement: S4 Fig — Representative images of immunofluorescence staining of ETs released by E. andrei coelomocytes collected from earthworms treated for 24 hrs with bacteria X. bovienii (BACT). Retrieved coelomocytes were seated in slide chambers and the immunostaining with anti-HSP27 antibody (green) was performed after 24 hrs; additionally, Sytox orange (red) was used to counterstain extDNA. Images presented in the right panel also visualize bacteria (red, stained with Sytox orange) binding to ETs composed of extDNA and HSP27 (merge images). Scale bar on all images 20 μm. (TIF) [file pone.0159031.s004.TIF]

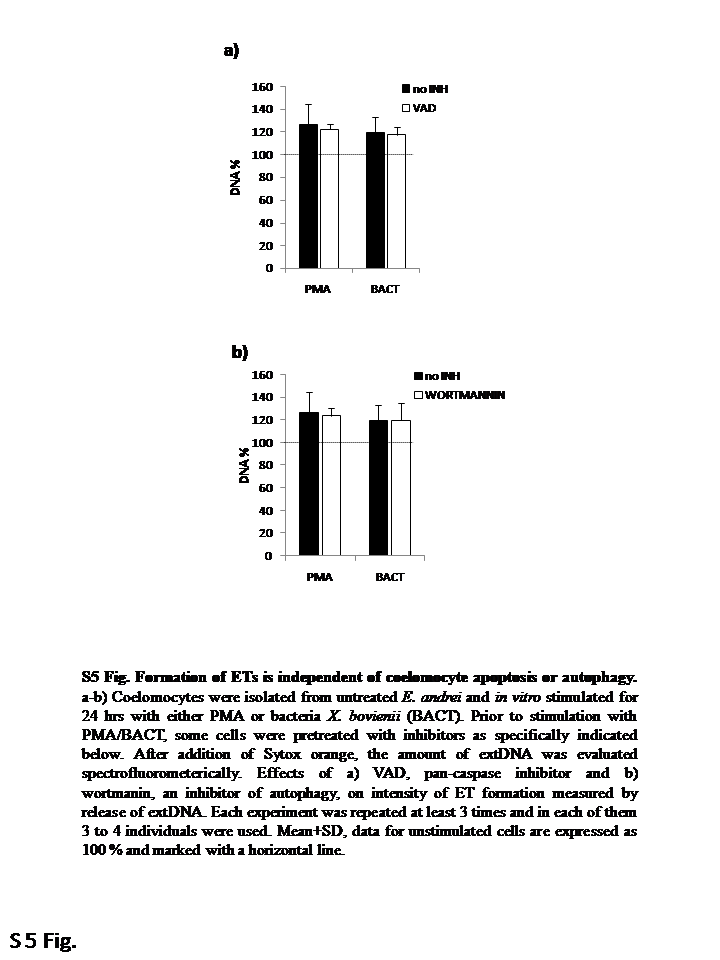

Supplement: S5 Fig — a-b) Coelomocytes were isolated from untreated E. andrei and in vitro stimulated for 24 hrs with either PMA or bacteria X. bovienii (BACT). Prior to stimulation with PMA/BACT, some cells were pretreated with inhibitors as specifically indicated below. After addition of Sytox orange, the amount of extDNA was evaluated spectrofluorometerically. Effects of a) VAD, pan-caspase inhibitor and b) wortmanin, an inhibitor of autophagy, on intensity of ET formation measured by release of extDNA. Each experiment was repeated at least 3 times and in each of them 3 to 4 individuals were used. Mean+SD, data for unstimulated cells are expressed as 100% and marked with a horizontal line. (TIF) [file pone.0159031.s005.TIF]
